# Molecular Pharmacology of Rosmarinic and Salvianolic Acids: Potential Seeds for Alzheimer’s and Vascular Dementia Drugs

**DOI:** 10.3390/ijms19020458

**Published:** 2018-02-03

**Authors:** Solomon Habtemariam

**Affiliations:** Pharmacognosy Research Laboratories & Herbal Analysis Services, University of Greenwich, Central Avenue, Chatham-Maritime, Kent ME4 4TB, UK; s.habtemariam@herbalanalysis.co.uk; Tel.: +44-208-331-8302 (ext. 8424)

**Keywords:** Alzheimer’s disease, amyloid beta, caffeic acid, danshensu, dementia, neurodegeneration, rosmarinic acid, salvianolic acids, tau protein

## Abstract

Both caffeic acid and 3,4-dihydroxyphenyllactic acid (danshensu) are synthesized through two distinct routs of the shikimic acid biosynthesis pathway. In many plants, especially the rosemary and sage family of Lamiaceae, these two compounds are joined through an ester linkage to form rosmarinic acid (RA). A further structural diversity of RA derivatives in some plants such as *Salvia miltiorrhiza* Bunge is a form of RA dimer, salvianolic acid-B (SA-B), that further give rise to diverse salvianolic acid derivatives. This review provides a comprehensive perspective on the chemistry and pharmacology of these compounds related to their potential therapeutic applications to dementia. The two common causes of dementia, Alzheimer’s disease (AD) and stroke, are employed to scrutinize the effects of these compounds in vitro and in animal models of dementia. Key pharmacological mechanisms beyond the common antioxidant and anti-inflammatory effects of polyphenols are highlighted with emphasis given to amyloid beta (Aβ) pathologies among others and neuronal regeneration from stem cells.

## 1. Introduction

Epidemiological and global impact analysis of dementia has been regularly updated by the World Health Organization and various institutions associated with the disease such the Alzheimer’s societies. According to the Alzheimer’s Disease International [1], dementia is one of the leading public health priority with a global prevalence of 46.8 million that is also expected to double every 20 years or a projection of around 131.5 million cases by 2050. The disease is associated with progressive cognitive impairment and function requiring continuous care and often institutionalization.

Accounting for around 70% of all cases, the leading cause of dementia is Alzheimer’s disease (AD) [1,2]. Most often presented as an age-related disease, the global prevalence of AD has seen rapid increase in parallel with increased life expectancies resulting from progressive economic development. The second most leading cause of dementia in the world is cerebrovascular disease (CVD) with up to one third of stroke survivors are estimated to suffer from some form of dementia or post-stroke dementia [3]. Mixed dementia is a case where AD and CVD occur together while numerous other causes including long-term cognitive disorder associated with alcoholism [4], epilepsy [5], etc. have been reported.

**Figure 1:**
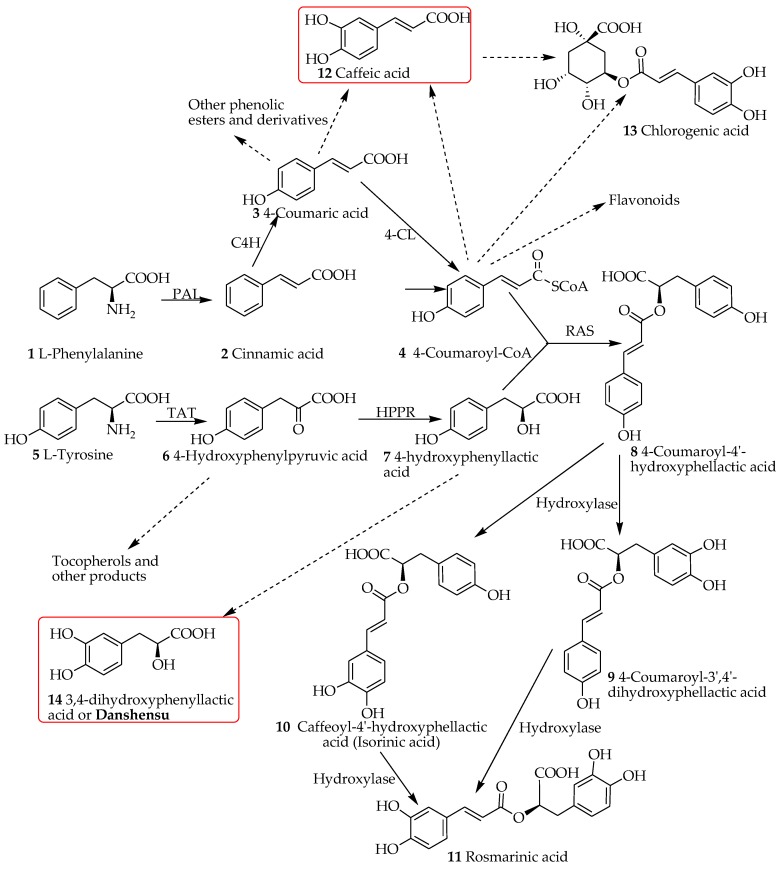
Overview of the biosynthesis pathway of rosmarinic acid and related compounds in plants. 4CL, hydroxycinnamate (or 4-coumaric acid) coenzyme A ligase; C4H, cinnamic acid 4-hydroxylase; HPPR, hydroxyphenylpyruvate reductase; PAL, phenylalanine ammonia-lyase; RAS, rosmarinic acid synthase; TAT, tyrosine aminotransferase. Compounds highlighted by red box are precursors of the salvianolic acids. Dashed arrows represent multi-step reactions.

The lack of real progress in dementia therapy is evident from the existing therapeutic approaches that all put their focus in reducing the progressive clinical symptoms instead of cure. Thus, a great deal of global efforts has been placed to look for potential drugs from both synthetic and natural sources that offer novel approaches or unique molecular targets. Beyond our own input in this search efforts, review articles from our laboratories have scrutinized the anti-AD potential of numerous natural products in general [6] and specifically those belonging to the class of monoterpenes (e.g., the iridoids class) [7], diterpenes [8] and flavonoids [9,10,11,12,13]. The other natural product that we highlighted as potential therapy for dementia was caffeic acid along with its ester derivatives such as chlorogenic acid and caffeic acid phenethyl ester [14]. The fairly few steps in the biosynthetic pathway of caffeic acid and related compounds (see Figure 1) and its ubiquitous presence in our common food ingredients such as coffee, cherries, apples and honey give us even more incentive to investigate these compounds as potential leads. The present review is designed to investigate the molecular and gross pharmacological profile related to dementia of compounds called salvianolic acids and rosmarinic acid (RA). Structurally, these compounds are composed of caffeic acid (structure **12**) and a related phenolic compound, danshensu (3,4-dihydroxyphenyllactic acid, structure **14**, Figure 1).

## 2. Natural Occurrence and Biosynthesis of Rosmarinic and Salvianolic Acids

Rosmarinic acid (RA, stricture **11**, Figure 1) was first isolated from *Rosmarinus officinalis* and characterized by Italian scientists in 1958 [15]. The natural distribution of RA has been widely reported. In a review by Petersen and Simmonds [16], plants belonging to at least 10 families of the dicots and five monocots were reported to synthesize RA. Members of the family Lamiaceae predominantly reported as common herbal sources of RA include common sage (*Salvia officinalis*), peppermint (*Mentha piperita*), thyme (*Thymus vulgaris*), lemon balm (*Melissa officinalis*) and Rosemary (*R. officinalis*). The phylogenic relationship within the angiosperms with respect to RA biosynthesis as well as detailed prevalence in the plant kingdom has been reviewed by Petersen et al. [17].

Petersen et al. [17] have also outlined the biosynthesis of RA and its significance from evolutionary perspectives. They have shown the key enzymes involved in the biosynthesis machinery which by and large is the common shikimic acid pathway involved in the flavonoids and many other phenolic compounds’ synthesis in plants. The biosynthesis of RA involves two synthetic routes to yield the two monomeric units, caffeic acid (**12**) and danshansu (**14**) that are both products of the shikimic acid pathway. Without going into details, one can start these biosynthesis steps from the primary metabolites, phenylalanine (**1**) and tyrosine (**5**, Figure 1). The deamination of phenylalanine through the action of phenylalanine ammonia-lyase (PAL; E.C. 4.3.1.5) results in cinnamic acid (**2**). Further catalysis by cinnamic acid 4-hydroxylase (cytochrome P450 monooxygenase cinnamate 4-hydroxylase (CAH; E.C. 1.14.13.11) yields *p*-coumaric acid (or 4-coumaric acid, **3**) that is widely distributed in plants in various forms including the lingnins/lignans. The transformation of this compound by hydroxycinnamate coenzyme A ligase (4CL; E.C. 6.2.1.12) yield 4-coumaroyl-CoA (**4**) that serves as a precursor to flavonoids as well as RA (**11**) biosynthesis. In another route of biotransformation, tyrosine aminotransferase (TAT; E.C. 2.6.1.5) acts as an entry point enzyme [18] and converts tyrosine (**5**) to the phenylpyruvate derivative (**6**) that is reduced by the action of 4-hydroxyphenylpyruvate reductase to give 4-hydroxyphenyllactic acid (**7**); a precursor of danshensu (**14**). The coupling of compound **7** with 4-coumaroyl-CoA (**4**) is an esterification process catalyzed by rosmarinic acid synthase (RAS). Oxidation of the aromatic rings can then proceed to yield various products including RA (**11**) which carries a catechol functional group at both ring systems. This process, as outlined in Figure 1 can take one step at a time in both rings leading to RA (**11**) and the presence of compounds like isorinic acid (**10**) in nature shows this transition process. Hydroxylation at a specific C-3 position of the aromatic skeleton to give these two intermediates have also been suggested: i.e., the existence of 4-coumaroyl-4′-hydroxyphenyllactate 3/3′-hydroxylases and caffeoyl-4′-hydroxyphenyllactate 3′-hydroxylase enzymes in RA producing plants [17]. These two distinct routes of caffeic acid and 3,4-dihydroxyphenyllactic acid (danshensu) in rosmarinic acid synthesis has been confirmed through numerous other studies since the 1970s in both plants and tissue culture studies [19,20]. Caffeic acid also exist in a range of other metabolites such as esters and glycosidic forms. Of the biologically significant caffeic acid derivatives are caffeic acid phenethyl ester and chlorogenic acid (**13**) and other quinic acid derivatives that display a plethora of pharmacological effects including anti-AD potential [14].

**Figure 2:**
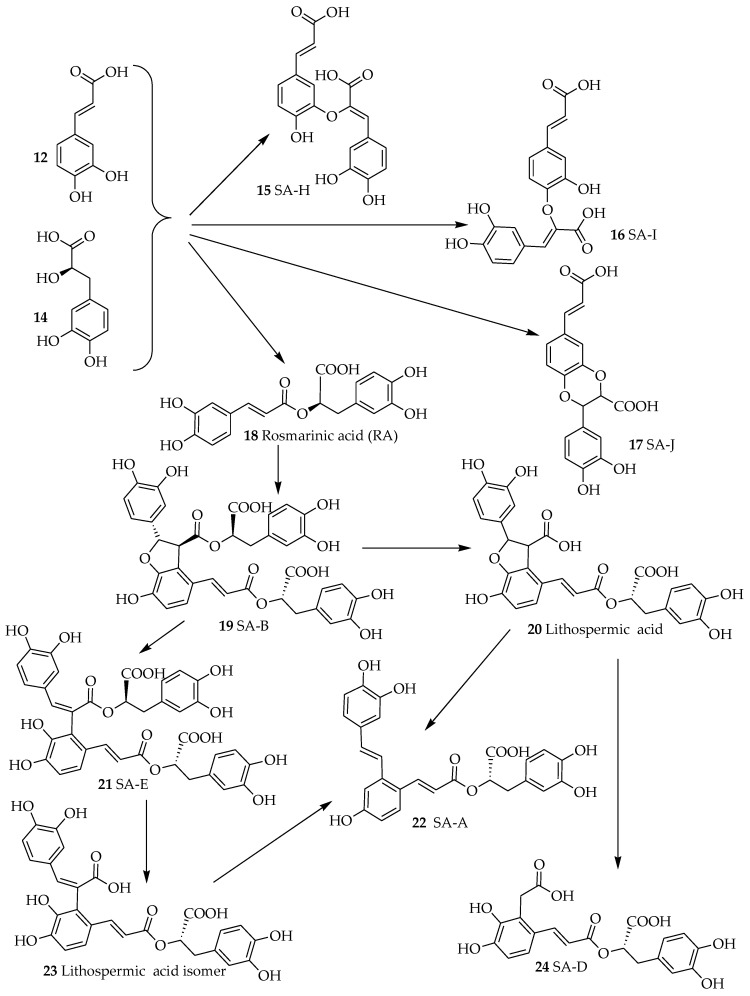
The routes of synthesis of salvianolic acids and related compounds. Following the synthesis of SA-B as dimeric RA, several oxidation and degradation reactions lead to diverse intermediates. Note that various stereoisomeric derivatives can also be obtained.

The other RA analogues are where the combination between caffeic acid (**12**, Figure 2) and danshensu (**14**) occur via esterification at other sites. Classical examples are salvianolic acid H (SA-H, **15**), I (SA-I, **16**) and J (SA-J, **17**). The major structural diversity of RA however comes when two units of RAs are combined to form a compound as that represented by salvianolic acid B (SA-B, **19**). This compound appears to be a precursor to many related salvianolic acid derivatives and lithospermic acids (Figure 2). The interconversion of these compounds has been studied by various authors. Li et al. [21] with the review of previous studies have outlined degradation processes in plant tissues and aqueous extracts where salvianolic acid A (SA-A, **22**) is shown as the degradation product of SA-B among others (Figure 2). Other studies have further shown that such transformation can readily takes place under high temperature, high pressure and high humidity environments in aqueous media [22]. Today, the acquisition of these compounds through total synthesis has become a common practice. Zheng et al. [23] have reported an 8-step synthesis of (+)-SA-A in a yield of 10.6%. Fortunately, this synthesis process does not have to go through the long synthesis and degradation pathway of SA-B in living system. Other synthesis approaches of salvianolic acid derivatives have also been postulated [24,25,26]. From the biological point of view, both for purified compounds and plant-based drug preparations, SA-A and SA-B are the most important and hence emphasis is given in this review to highlight their effect in the dementia brain. Both compounds are extracted from the root of *Salvia miltiorrhiza* Bunge (Labiatae) (“Danshen” in Chinese, also known as Chinese sage): SA-B is the most abundant and bioactive of the salvianolic acids [27]. The plant material is also extensively used not only in the traditional Chinese medicine but as danshen dripping pills (a mixture of *Salvia miltiorrhiza*, notoginseng, and borneol) which have been the subject of numerous clinical trials in recent years [28].

## 3. Salvianolic Acids and Dementia

### 3.1. In Vitro Effects

**Figure 3:**
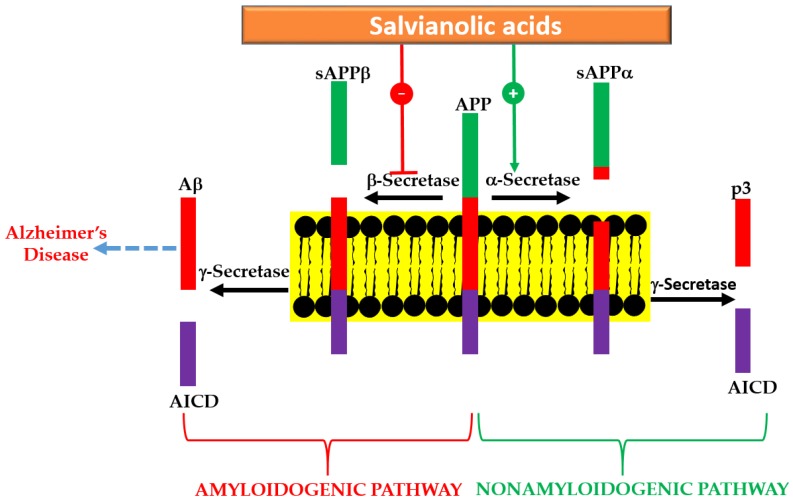
Anti-AD effect of salvianolic acids via Aβ formation. In the amyloidogenic pathway, APP is first hydrolyzed by BACE1 and generates sAPPβ and C-terminal fragment-β of APP (CTF-β). γ-Secretase further cleaves CTF-β to release APP intracellular domain (AICD) and Aβ, which aggregates to form amyloid plaques. On the other hand, the nonamyloidogenic pathway involves the cleavage of APP by α-secretase to release sAPPα and CTF-α. The latter is further cleaved by γ-secretase to yield two fragments p3 and AICD. The effect of salvianolic acid via modulation of both pathways is shown.

A summary of in vitro effects that attributes to the potential therapeutic effects of salvianolic acid derivatives are shown in Table 1 [29,30,31,32,33,34,35,36,37,38,39,40,41,42,43,44,45]. By assessing the β-secretase (β-site amyloid precursor protein (APP) cleaving enzyme or BACE1) and γ-secretase inhibitory activity, a selective effect against BACE1 was demonstrated for SA-B [32]. Moreover, the direct interaction of the compound with the catalytic center of the enzyme has been confirmed by docking experiments [32]. In SH-SY5Y-APPsw cells, the Aβ40 and Aβ42 levels in culture media has been shown to be suppressed by SA-B [33]. While the protein expression of APP was not affected by SA-B, it has been shown to increase the protein level expressions of “a disintegrin and metalloproteinase domain-containing protein 10” (ADAM10) and secreted APP-α (sAPPα) concomitantly with the decreased protein expressions of BACE1 and sAPPβ [33]. Such observation is in perfect agreement with the postulated mechanism of action via inhibition of Aβ formation (Figure 3). The nonamyloidogenic pathway via the ADAM10 as a classic example of α-secretase within the Aβ domain that breaks down APP has now been well established [46,47]. Interestingly, SA-B has been reported to increase the activity of α-secretase while suppressing β-secretase. Hence, an augmented α-secretase pathway which is nonamyloidogenic and a decrease in Aβ generation via amyloidogenic pathway was evident for SA-B (Figure 3).

Once Aβ is released, its self-aggregation to form fibrils is a prerequisite to induction of neurotoxicity in the AD/dementia. Hence, compounds that either inhibit Aβ aggregation or those promoting disaggregation of preformed fibrils are expected to have anti-AD potential. The effect of SA-A in reducing the metal-induced aggregation by chelating metal ions; block the formation of intracellular reactive oxygen species (iROS); inhibition of Aβ self-aggregation through binding to the C-terminus and hence stabilizing the helical conformations, was a finding by Cao et al. [31] worth mentioning. The experiment by Durairajan et al. [38] also shed some light into the mechanism and level of potency of SA-B in Aβ aggregation/disaggregation. They have shown that the compound can ameliorate fibril aggregation with IC_50_ values between 1 and 5 µM while the destabilization effect on preformed Aβ_1–40_ fibril were similarly around 5 µM. This kind of activity in both the formation and the destabilization of Aβ fibrils have demonstrated for other natural products such as curcumin that have been shown to have a promise in AD [38].

Inhibition of cytotoxicity in neuronal cells induced by Aβ is another well-defined mechanism of therapeutic agents. The effect of SA-B in PC12 cell death induced by Aβ_25–35_ [34,37] is a classic example where cell survival coupled with reduction in ROS and intracellular Ca^2+^ (iCa^2+^) levels were observed. Another cellular model was the SH-SY5Y cells cytotoxicity induced by Aβ_1–40_ where protective effect was reported for SA-B. Worth noting is also the impressive level of potency where activity higher than 1 µM were shown to display cytoprotective effects.

When the pheochromocytoma cell line PC12 cells were exposed to H_2_O_2_-induced toxicity, SA-B has been demonstrated to display protective effect at concentrations less than 10 µM [39]. In addition to reducing the oxidative stress level as shown by the level of malondialdehyde (MDA) level, increased antioxidant status (enhanced activities of superoxide dismutase (SOD), catalase (CAT) and glutathione (GSH)-peroxidase (GPx)) was shown. Hence, the compound, beyond the known direct radical scavenging effect against ROS, could enhance the antioxidant defence in neuronal cells. The suppression on iCa^2+^ and caspase-3 activity is also in line with anti-apoptotic effect of this compound in neuronal cells [39]. Another model of significance both in vitro and in vivo is the oxygen-glucose deprivation/reperfusion (OGD/R) damage in neurons. The data by Wang et al. [30] on primary rat cortical neurons showed that SA-B is cytoprotective primarily by increasing the activities of antioxidant enzymes such as Mn-SOD, CAT and GPx. The mitochondrial mechanism of cell death induced by the release of cytochrome c and induction of apoptosis was also demonstrated from protective effects on mitochondrial membrane potential (ΔΨ(m)). Similar mechanisms and protective effects were reported for salvianolic acid (undescribed) of commercial source [40].

Using the OGD/R model of cell damage in PC12 cells, Wang et al. [35] also showed the protective effect of SA-B through anti-inflammatory mechanism. Hence, the common inflammatory target, NF-κB, transcriptional activity and pro-inflammatory cytokine responses (IL-1β, IL-6, and TNF-α) have been shown to be suppressed. The suppressive effect of SA-B in LPS-stimulated primary microglial cells is a further example of anti-inflammatory effects [29]. This anti-inflammatory mechanism is elaborated in great detail in Section 5.

Incredible level of attention has also been given in recent years to evaluate the potential of salvianolic acids in promoting neuronal cell growth following ischemic or other forms of CNS pathology. Interestingly, SA-B has been shown neuronal cell growth and differentiation from oligodendrocyte precursors [36,41]. When bone marrow derived neural stem cells were treated with SA-B, an enhanced potential for self-renewal and neuronal differentiation have been observed while cell survival, including under oxidative stress condition induced by H_2_O_2_, were augmented by the compound [36]. Moreover, the induction of brain-derived neurotrophic factor (BDNF) production by SA-B in these cells was in line with potential therapy not only to ameliorate the accelerated neuronal cell death in dementia but also their recovery from stem cells [41]. In a similar experimental model, induction of cellular proliferation and self-renewal maintenance by SA-B was coupled with upregulated expression of nestin (marker protein of neuronal stem cells) via the PI3K/Akt pathway [42]. All these effects also appeared to be mediated at concentrations from 5 to 50 µM and include promotion of neurite outgrowth and their differentiation into neurons [44].

**Figure 4:**
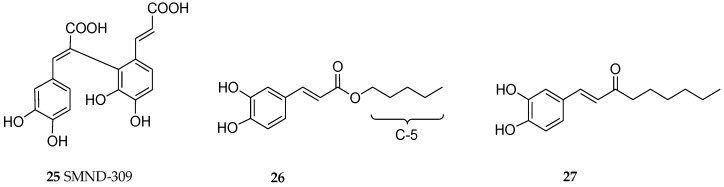
Structures of some synthetic analogues with proven biological effects related to dementia. SMDD (**25**) is a caffeic acid dimer that does not involve an ester bond but rather a C-C bridge. The C-5 chain derivative, compound **26**, was found to have an optimum structure in antioxidant and Aβ aggregation inhibition studies from studies on derivatives including C-3 to C-9 chain length, while compound **27** was a prototype lead compound that does not have an ester linkage.

Most of the in vitro studies on neuronal cells so far appear to focus on SA-B, the most predominant component of the natural salvianolic acids in the crude drug preparation of *S. miltiorrhiza*. An insight into the synthetic source of compounds with similar mode of action has been the subject of many studies too. For example, SMND-309 (Figure 4) is a caffeic acid dimer which has been shown to display similar effect in the OGD/R model in vitro (Table 1). Other effects of SA-B in vitro is associated with increased antioxidant status in neuronal cells. Given the structural moieties including caffeic acid and/or catechol functional groups, radical scavenging effects (e.g., [48]) is expected for these compounds. The effect of SA-B in suppressing the cardiotoxicity of doxorubicin is also mediated through antioxidant mechanism [49]. SA-B could also inhibit GSK3β by increasing the ratio of pSer9-GSK3β to total GSK3β [33].

### 3.2. Salvianolic Acids Ameliorating Dementia in Animal Models

The various effects of salvianolic acids in AD and CVD animal models are summarized in Table 2 [50,51,52,53,54,55,56,57,58,59,60,61,62,63,64,65,66,67,68,69]. The data by Shen et al. [52] in transgenic mice model show not only improvement of learning and memory coupled with a reduction of Aβ level, but also various other metabolic markers. This include the reduction in plasma low-density lipoprotein cholesterol (LDL-C) level which appeared to be positively correlated with Aβ_1–42_ level in the hippocampus. The positive correlation between Aβ and LDL-C or negative relationship between Aβ and high density lipoprotein (HDL) is in line with lipid lowering and cardiovascular protective effects [70,71]. Cholecalciferol (vitamin D) is expected to have an enhanced function in Aβ clearance and its reduced level by salvianolic acids is expected as Aβ level is normalized [51]. The data by Lee et al. [64] also provided the direct effect of SA-B in Aβ-induced memory loss and inflammation in vivo.

The critical role of glial cells (primarily astrocytes and microglial cells) both in orchestrating the Aβ induced inflammation and neurotoxicity in animals, and their protective effects in the Alzheimer’s brain have been well established [72]. The crosstalk between this inflammatory cascade and ROS are also evident as the latter could also augment the inflammatory response [73]. While glial cells are involved in the clearance of Aβ, their overactivity in AD pathology is known to exacerbate AD and hence they are serving as drug targets for therapeutic intervention. The data by Lee et al. [64] showing a suppressive effect on cyclooxygenase 2 (COX-2) and inducible nitric oxide synthase (iNOS) by SA-B along with suppression of glial activation and memory improvement is a good set of in vivo evidence of efficacy. One must also note the low dose employed (10 mg/kg) via the oral route of administration. The direct effect of SA-B in AD model also came from studies where Aβ_25–35_ was injected into animals. The resulting behavioural changes that could be ameliorated by SA-B appeared to be mediated via the γ-aminobutyric acid (GABA)-ergic neurotransmitter system [66]. Through such mechanism, the effect of SA-B in reversing the scopolamine and other drugs-induced cognitive impairments have been established [66]. More specifically, SA-B (100 μM) was found to inhibit GABA-induced outward Cl^−^ currents in single hippocampal CA1 neuron [66]. In a similar model in mice, improvement of learning and memory could be achieved by SA-B along with inhibition of glial activation and reduced level of inflammatory markers such as iNOS and COX-2 expression levels as well as oxidative stress [64].

In view of the well-recognized pharmacological effects of salvianolic acids and the plant that produce them, *Salvia miltiorrhiza*, several studies have been directing their focus on neuroprotective effects in ischemia/reperfusion injury model in animals. The study by Fan et al. [50] highlighted that the protective effect of SA-B was coupled with suppression of ROS and pro-inflammatory cytokines (IL-1β, IL-6 and TNF-α). Through such action, the activation of glial cells could be suppressed by this compound. The data by Zhang et al. [29] is also in agreement where microglial activation or M1 microglial polarization toward M2 is targeted by SA-B (Table 2). Other studies with the same anti-inflammatory mechanism for SA-B are also reported [60,62]; while others show augmented antioxidant defences such as SOD level/activity along with suppressed MDA or ROS levels [61,69]. The ischemia/reperfusion (I/R) injury model study in rats also revealed that SA-B via the intraperitoneal route not only suppress neuronal deficits at small doses but also ameliorate the inflammatory components as evidenced from cytokines and adhesion molecules expression (Table 2). A commercial product called “salvianolic acids for injections”, which predominantly contain SA-B, has also been shown to have similar effect on neuroprotection and neuroinflammation in vivo [57]. Numerous other studies also reported the same findings for SA-B in this model [35,40]. More importantly, memory functions in vascular dementia has been shown to be prevented by SA-B [42].

The effect of SA-B in other models include where direct physical damage to central neurons is induced by heavy impact on the spinal cord. As shown by Zhu et al. [36], SA-B could promote myelin sheath recovery and the number of regenerating axons while gross neurological function was also recovered. These effect of SA-B could in part be explained by enhancing the expression of anti-inflammatory cytokines (IL-10) while suppressing pro-inflammatory cytokines such as TNF-α and IL-1β [67]. Such an effect was also shown to be linked with improvement in spatial learning and memory [67].

Salvianolic acid A was also tested in cerebral ischemia model where neuroinflammation and peroxynitrite (ONOO^−^) generation were shown to be suppressed [57]. Furthermore, upregulation of the protein kinase B (PKB or Akt), Forkhead box protein O1 (FKHR or FOXO1) and extracellular signal–regulated kinases (ERK) phosphorylation were reported in these experiments [57]. One remarkable observation was also the rather small intraperitoneal effective doses reported (1 mg and 5 mg). The neuroprotective effect SA-A in blood-spinal cord barrier (BSCB) in spinal cord injury model in rats has also demonstrated at small doses (2.5, 5 and10 mg/kg, i.p.) which were also coupled with the expression of antioxidant marker proteins such as erythroid 2-related factor 2 (Nrf2) and heme oxygenase-1 (HO-1) [58]. As with SA-B, the effect of SA-A on neuroprotection in vivo through reduced oxidative stress and anti-inflammatory mechanisms including the suppression of key inflammation cytokines (TNF-α, IL-1β, IL-6, and IL-8) have been demonstrated [52]. Moreover, the compound reversed the increased level of phosphorylation of p38 mitogen-activated protein kinase (MAPK) and the decreased level of phosphorylation of ERK induced by subarachnoid haemorrhage in rats. This compound could also offer protective effect in ischemic brain injury model in mice when administered at 50 and 100 μg/kg, i.v. [59]. The neuroprotective effect was also coupled with reduced level of oxidative stress and anti-inflammatory effects via downregulating the NF-κB pathway [59]. In transgenic *Caenorhabditis elegan* model, Aβ-induced paralysis could also be inhibited by SA-A [31]. The effect of SA-A in brain protection from haemorrhage via suppression of oxidative stress and by upregulating the Nrf2 antioxidant mechanism have been established [52]. Several other experiments also showed that SA-A via activation of the Nrf2 signalling pathway ameliorate oxidative stress under cellular and pathological conditions such as diabetes [74,75]. All the data presented in these studies were consistent with key inflammatory cytokine mediators such as IL-1β, TNF-α and IL-6 were suppressed suggesting the antiinflammatory effect of SA-A in the brain.

One common model of study frequently used to assess the central effect of salvianolic acids is the vascular dementia model in animals via transient or permanent occlusion the carotid artery. Ma et al. [53] have shown the potential of SA-B via the oral route of administration and a dose as small as 20 mg/kg. Moreover, the induction of apoptosis in hippocampal neurons through this model is inhibited by this drug via increasing the increased phosphorylated Akt level without altering the protein level of Akt. Following ischemic stroke, the process of angiogenesis and long-term neurological recovery have been studied by various authors. Li et al. [54], for example, have shown that the total salvianolic acid drug preparation could enhance angiogenesis and neuronal recovery through activation of the Janus kinase 2/signal transducer and activator of transcription 3 (JAK2/STAT3) signalling pathway. The synthetic drug SMND-309 that were shown some in vitro effect in neuroprotection has also been shown to display neuroprotection in various in vivo models [64,67,68]. The role of these potential therapeutic agents in combination with other drugs is also worth mentioning. For example, Yu et al. [76] have studied the combined effect of fluoxetine (20 mg/kg) and salvianolic acid (40 mg/kg) in rats under chronic stress. Treatment for three weeks with a combined approach has been shown to have a far better cognitive improvement in animals.

Various commercial products of these compounds are now available along with plant extracts that often contain SA-B as predominant marker. For example, chromatographic trace of salvianolic acids for injection as a commercial brand is predominantly SA-B (63.81%) containing trace amounts (5% or less) of rosmarinic acid, salvianolic acid D and Y among other trace compounds [56]. The therapeutic effect of crude drugs such as *S. miltiorrhiza* root preparations therefore need to be reflected in light of this reality. Most of the compounds such as SA-A and other derivatives are also active, however, and are likely to contribute to the overall effect of the crude drugs.

## 4. Rosmarinus Acid (RA) in AD

Several in vitro and animal studies have shown the promise of RA in AD therapy. The experiment by Taguchi et al. [77] using docking simulation and direct binding studies investigated the structural features of RA that allowed it to directly interact with Aβ_1–42_. They have shown that the catechol functional group on the caffeic acid side is an important structural feature for binding. On the other hand, the ester-bond on the danshensu side which could be liable to breakdown in vivo could be replaced by favourable chain-length substituents. Hence, they have shown two compounds (**26** and **27**, see Figure 4) that are sufficiently antioxidant as assessed by xanthine oxidase and DPPH radical-scavenging effects while at the same time showing good inhibition in Aβ_1–42_ aggregation.

The experiment by Espargaró et al. [78] also noted that the effect of RA in Aβ aggregation was similar with other natural products known for such effects including melatonin, *O*-vanillin, curcumin, apigenin and quercetin. In cultured PC12 cells treated with Aβ_1–42_, cytotoxicity coupled with ROS formation, lipid peroxidation, DNA fragmentation, caspase-3 activation, and tau protein hyperphosphorylation were all suppressed by RA (10 µM) [79]. On the other hand, evidence on the potential anti-AD effect of RA through Aβ antagonism has also been demonstrated in vivo. Doses as small as 0.25 mg/kg through oral route of administration for 14 days in mice were shown to ameliorate the effect of intracerebroventricularly (i.c.v.) injected aggregated Aβ_25–35_ [80]. The favourable outcomes include memory improvement along with decreased levels of NO and MDA levels in the brain, kidney and the liver [80]. Oral administration of RA (50 mg/kg) for 60 days to ovariectomized rats treated with d-galactose have also shown to restore the altered locomotor activity and cognitive functions. Furthermore, other favourable effects in oxidative stress (e.g., lipid peroxidation levels) and inflammation (COX-2 expression and PGE-2 levels) markers in brain tissue were observed [81]. In mice pre-injected (i.c.v.) with Aβ_25–35_, administration of a small dose of RA (0.25 mg/kg, i.p.) could ameliorate the nitration of proteins and cognitive abnormalities [82].

Cornejo et al. [83] have done a pioneering experiment to show the role of RA on tau protein precipitation and/or aggregation. They have shown that RA binding to tau protein in vitro leads to a decrease in amide regions I and III suggesting that the compound inhibits β-sheet assembly. Their molecular docking study further showed that RA binds to the steric zipper in the same manner as orange G. Interestingly, these activities were demonstrated at effective concentration of 10 μM where both fibril formation and progression to oligomers are inhibited. In addition to the in vitro data, the effect of RA as anti-AD agent in vivo through tau protein modulation has been demonstrated [84]. As expected, they found that the level of phosphorylated tau protein increase with age and chronic restraint stress (CRS) while inverse relationship was noted for the level of chaperones expression (i.e., diminished with age or stress). CRS was also shown to suppress the expression of Pin1, the peptidylprolyl *cis*/*trans* isomerase, in aging animals. All these parameters as well as phosphorylated tau protein and insoluble phosphorylated tau protein formation induced by stress/aging were shown to be revered by RA [84].

When tested at 10 μg/mL in vitro, RA has also been shown to display inhibitory effect against acetylcholinesterase (AChE) and butyrylcholinesterase (BChE) by 28% and 80% (IC_50_ = 6.59 μg/mL) respectively [85]. Hence, it was more active in BChE than AChE. In vitro and ex vivo studies as well as in silico docking studies by Demirezer et al. [86] also showed its AChE inhibitory activity along with its known antioxidant capacity. The anti-AChE activity of RA was also investigate by Szwajgier [87] in comparison with other phenolic acids where the order of potency was established in the following order: homogentisic acid > 4-hydroxyphenylpyruvic acid > nordihydroguaiaretic acid > rosmarinic acid > caffeic acid > gallic acid = chlorogenic acid > homovanillic acid > sinapic acid. Hence, RA appear to be more potent than one of its monomer structural component, caffeic acid. The potential of RA in memory enhancement potentially through AChE and BChE inhibition have also been established in many other studies [88,89].

## 5. Summary of Molecular Mechanisms That Attribute to the Dementia-Related Pharmacology of Salvianolic and Rosmarinic Acids

To date, the therapeutic approaches of dementia are limited to disease management and bring about some symptomatic relief. For the most prevalent form of dementia, AD, the pharmacological approach of therapy is mainly driven by boosting cholinergic output. Classical examples are cholinesterase inhibitors including donepezil, galantamine, and rivastigmine. The limited symptomatic relief by using non-competitive *N*-methyl-d-aspartate receptor antagonists (e.g., memantine) have also been clinically shown. It is also worth noting that attempts to find anti-AD therapy by abolishing the Aβ loads has so far not been successful and approaches employing multiple targets or novel mechanism are necessary (see Section 7). For detailed review on current therapeutic approaches, readers are directed to review articles in this field [90,91]. The application of salvianolic acids and RA appear to target not only biochemical mechanisms of AD but also CVD that is applicable to stroke. A summary of these targets at molecular level is presented in the following sections.

### 5.1. Direct Effect on Aβ Formation and Aggregation, and ROS Generation

A quarter of a century has now elapsed since the Aβ hypothesis of AD has been postulated to put Aβ formation and clearance dysfunction as the key pathological marker and/or therapeutic target. Numerous small molecular weight compounds including some common food ingredients and peptides modulators have also been identified and extensive review articles on the overall therapeutic principle have been published (e.g., [92,93,94]). Interestingly, Aβ appear to be the main molecular target for salvianolic acid and RA derivatives and the whole cascade of Aβ formation from APP, aggregation and toxicity to neuronal cells are ameliorated by these compounds. With respect to Aβ formation, the key amyloidogenic pathway enzyme, β-secretase (BACE1), has been reported to be suppressed by SA-B and analogues while the nonamyloidogenic pathway enzyme ADAM10 (α-secretase) was augmented (e.g., [33]). In animal model of AD, the level of Aβ has also been suppressed by these potential therapeutic agents (e.g., [51]). Hence, one possible therapeutic target is Aβ formation as depicted in Figure 3.

One of the hallmark of AD is the extracellular precipitation and accumulation of aggregated Aβ that contributes to the observed biochemical and behavioral symptoms primarily through induction of neuronal cell death. The role of metal ions such as copper, zinc and iron in orchestrating the Aβ aggregation and toxicity through the generation of ROS have been well established. Hence one of the therapeutic strategy in AD could come from overcoming the metal-induced Aβ pathology [95,96,97]. Hand-in-hand with the high level of transition metal ions associated with the Alzheimer’s brain, ROS-induced neuronal damages has been shown to be correlated with Aβ pathology [98,99]. The severity of AD pathology has also been shown to be inversely correlated with the level of antioxidant defenses (both small molecular weight and proteins) in the brain [35,36,37,38]. Moreover, mitochondrial dysfunction associated with ageing are the common source of ROS that contributes to AD severity [39]. Not surprisingly, salvianolic acids and RA that are efficient radical scavengers are also shown to boost the antioxidant defenses in cellular and animal models (see Table 1 and Table 2). They also ameliorate the aggregation of Aβ that is a prerequisite to its toxicity in neuronal cells. One critical structural feature for these compounds both in inhibition of Aβ aggregation and ROS-mediated toxicity is the catechol functional moiety that comes in these compounds in good number (Figure 2). The association between this functional group and anti-AD effects has been established in RA and analogues [77]. We have also shown that such functional group in phenolic acids as well as in the flavonoids skeleton are optimized for numerous pharmacological effects linked to ROS, metal ions chelation and enzyme inhibition [100,101,102,103,104,105,106,107,108,109,110,111,112,113,114,115,116,117,118,119]. Hence, the binding of SA-B with serum albumin was also suppressed in the presence of Zn^2+^, Cu^2+^, Co^2+^, Ni^2+^ and Fe^3+^ suggesting direct interaction with the drug [120]. In a previous review from this laboratory, the coordination of metal ions with the catechol structural moiety and such chelative effect as mechanism of biological activity in ROS generation and toxicity have been outlined [14]. Interestingly, loss of memory function and learning by chronic metal (e.g., aluminum) intoxication could be reversed by compounds such as caffeic acid [121]. Hence, the observed effect of salvianolic acids and RA derivatives in dementia could be in part explained by the well-known therapeutic strategy of ameliorating the ROS-Aβ-neurotoxicity crosstalk via redox metals (copper, zinc, and iron) coordination [98,99,122,123,124,125,126].

Salvianolic acids and other RA derivatives also appear to target the inflammatory pathway associated with the Alzheimer’s brain. The glial cells in the brain, primarily astrocytes and microglial cells, are involved in the normal tissue defense and homeostasis mechanisms but their overactivation has also been linked to AD pathology [127,128,129,130,131]. As shown in Table 1 and Table 2, various inflammatory mediators such as cytokines (TNF-α, IL-1β, and IL-6), COX and NOS as well as their products such PGE-2 and NO, respectively have been shown to be suppressed by these compounds. More importantly, key transcription factors such NF-κB that are associated with proinflammatory proteins expression are targeted. Hence, the observed effect is in line with therapeutic strategies where TNF-α or NF-κB are targeted in AD [132,133,134,135]. The crosstalk between inflammation and oxidative stress is also well-known and key mediators such as Nrf-2 and HO-1 that are modulated by these drugs (Table 2) lay down the foundation for possible molecular mechanisms of action.

**Figure 5:**
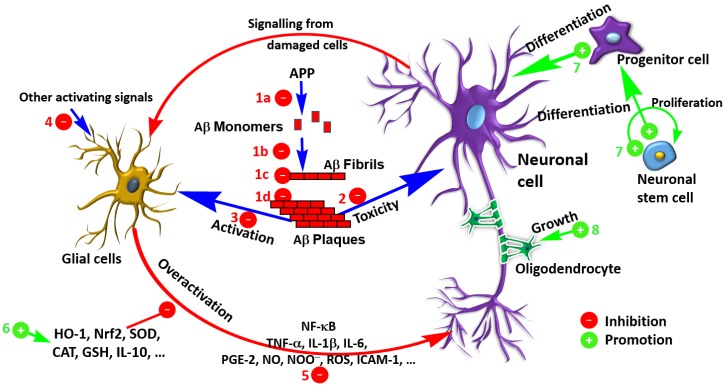
Overview of the therapeutic potential of salvianolic acids and RA derivatives in dementia via the various mechanisms of Aβ pathology. Mechanisms include inhibition of Aβ formation (1a), fibril formation/elongation (1b), interaction with fibrils (1c) and aggregated plaques (1d), toxicity in neuronal cells (2) and glial activation (3). The activation of glial cells to initiate inflammatory cascades (5) by various agents including other toxicants such as ROS (4) could be inhibited through processes including induction of antioxidant defenses (6). Other established mechanisms are neuronal regeneration from stem cells (7) and axonal and myelin sheath protection (8).

The law level of Aβ observed after treatment with salvianolic acid derivatives could also be attributed to increased clearance. More research is however required to show the contribution of such mechanism of action to the overall anti-AD effects in vivo. Several terpenoids such as iridoids have been shown to enhance Aβ by modulating the insulin degrading enzyme, the main extracellular Aβ degrading protease enzyme [7]. The role of the microglia in Aβ clearance is also known (e.g., [136,137]) but whether such mechanism makes significant contribution to the anti-AD effects of salvianolic acids and RA derivatives remains to be established. The overall mechanism of these compounds in ameliorating the Aβ toxicity through various mechanisms from direct effect on Aβ to general antioxidant and anti-inflammatory effects is shown in Figure 5.

### 5.2. Cholinesterase Inhibition

Given that AD is primarily linked to the loss of cholinergic neurons in the cortex, the major therapeutic strategy is linked to enhancing the activity of the surviving neurons. Based on this cholinergic hypothesis of the disease [138,139,140,141], AChE inhibition remains one of the major target for anti-AD drugs. The various mechanisms listed above for salvianolic acids and derivatives that augment the antioxidant status, ameliorate Aβ formation and toxicity, etc., inevitability lead to a better cholinergic neurons profile in animal models (Table 2). Direct effect on the AChE has also been reported in the various models (Table 1 and Table 2) and should be listed as one mechanism of action for these natural products.

### 5.3. Tau Protein Phosphorylation and Precipitation/Aggregation

One mechanism that does not appear to be well-established for salvianolic acids is modulation of tau protein phosphorylation. The level of phosphorylation of this protein in neuronal cells through regulation by various kinase and phosphatase enzymes is important for the normal function of the protein in diverse physiological processes associated to the microtubules function. The intracellular tangles as the hallmark of AD resulting from tau hyperphosphorylation is therefore an important pharmacological target for potential drugs [142,143,144]. The inhibition of β-sheet formation and assembly by RA has been, however, well documented [83,84]. Hence, both extracellular (Aβ) and intracellular (tau) protein aggregation that are clinically seen as AD pathological markers are targeted by RA.

### 5.4. Neuronal Regeneration Mechanisms

While maintaining the viability of surviving neurons and increasing their activity by approaches like AChE inhibition may offer some beneficial outcome in AD, the real breakthrough would be if the lost neurons are replaced through regeneration or recovery from stem cells. As shown both from cultured stem cells in vitro and animal experiments (Table 1 and Table 2), this established mechanism appears to be the most promising development in recent years for salvianolic acid derivatives [36,41,44]. The proliferation and differentiation of stem cells by this compound (Figure 5) is therefore one milestone to register. This mechanism also coincides with one of the best future hope of AD therapy, stem cell technology [145,146,147].

### 5.5. Cell Signalling

The MAPK include ERK, JNK, and p38 MAPK that are shown to play critical role in the regulation of diverse cellular functions including cell proliferation, differentiation, survival, inflammation and apoptosis [148]. Growth inhibition and induction of neuronal cell death can be induced by activation and phosphorylation of the p38 MAPK and JNK pathways. Several internal and external signals that induce oxidative stress (ROS) and induction of pro-inflammatory cytokines (e.g., TNF-α and IL-β) do also mediate their cell death signalling via activation of p38 MAPK and JNK [148]. More specifically, targeting the p38 MAPK pathway for the treatment of AD therapy has been advocated [149,150]. The role of these stress-associated kinases in tau protein phosphorylation and correlation with the level of neuroinflammation has also been described [151] and it is generally accepted that these kinases (JNK/p38) are considered as pathological markers of neuroinflammation in the Alzheimer’s brain. On the other hand, the ERK signalling has been shown to negatively regulate the β-secretase expression [152]. Activation of ERK and the PI3K signalling has also been shown to be associated with neuronal survival. Hence, the neuroprotective effects of some flavonoids as anti-AD principles have been shown to be partly mediated through activation of this pathways [153]. In this connection, various studies have shown that the neuroprotective effects of salvianolic acids is mediated via modulating the MAPK system (Table 1 and Table 2). For example, SA-A was reported to suppress the phosphorylation of p38 MAPK while increasing ERK phosphorylation [52,57]. The role of the GSK3β and PI3K/Akt pathways in the various neuronal processes including tau protein phosphorylation has been extensively reviewed [7,150,154,155]. The above-mentioned pharmacological effects of salvianolic acids and RA derivatives could thus be attributed to modulation of key signalling pathways.

## 6. Potential Drug Leads or Just Another Story of Diverse Pharmacological Effects by Polyphenols?

The occurrence of caffeic acid in common food ingredients and their diverse pharmacological effects have always been the subject of curiosity in both drug discovery and nutraceutical research. Caffeic acid, most commonly appearing as quinic acid esters such as chlorogenic acid and dicaffeoyl derivatives have also been shown to possess numerous pharmacological effects including antiviral activity and many others far beyond their common antioxidant effects [156]. One advantage of these compounds is their solubility in water and hence a better bioavailability through oral routs as shown from their efficacy in animal studies (Table 2). Several bioavailability studies on salvianolic acid derivatives have also been carried out to demonstrate their efficacy through an oral route. Formulations specifically for injection route such as “salvianolic acid for injection” are also available from which rosmarinic acid, SA-D, lithospermic acid and SA-B have been shown rapid distribution in rat tissues [157]. Studies on SA-B following nasal administration has also shown that the compound can reach the brain at a slower rate than the i.v. route but with prolonged and sustained rate [158]. Under I/R conditions, the cerbroprotective effect of SA-A has also been shown to be associated with more access to the brain than in normal animals [159]. Furthermore, various studies highlighted in Table 2 show that the effects of these compounds are also linked to restoring the BBB or BSCB that are consistent with their (or their active metabolites) entry into the brain. Worth mentioning is also their known prescribed effect as modulators of the cardiovascular system including stroke; the second most risk factor for dementia in humans. Hence, these compounds indeed have far more potential as therapeutic agents for dementia than just being seen as therapeutic leads. Moreover, the doses that have shown neuroprotective effect in the various animal models of dementia have been very impressive. From the traditional medicine point of view, the plant materials often in combination with other herbs, as demonstrated in the Chinese practice, are utilized as systematic medicine. Another pharmacological effect of these compounds worth mentioning is their potential application in drug combination as demonstrated for fluoxetine-SA-A combination [76].

As always, the promise of compounds established through in vitro and animal studies must be supported by clinical evidence to demonstrate the ultimate efficacy and safety for use in human system. In this regard, the hard lesson learnt from Aβ clinical trial is worth mentioning. Of the spectacular failure story reported recently was the discontinuation of the BACE inhibitor verubecestat from the Phase III clinical trial by Merck [160]. Even though some clinical trials based on Aβ approach are still ongoing (e.g., Eli Lilly), other setbacks from clinical trial such as bapineuzumab (Pfizer) and solanezumab (Eli Lilly) are grim reminders that in vitro and animal studies of the Aβ inhibitors may not correlate with efficacious under clinical conditions. According to ClinicalTrial.gov, danshen dripping pills (Identifier no.: NCT02388984) is currently under Phase III clinical trial for non-proliferative diabetic retinopathy. Clinical trial involving 200 participants for coronary heart disease and essential hypertension is also in active phase study in China (Identifier no.: NCT01825759). Other clinical trial mentioned in ClinicalTrial.gov are danshen extract for cardiovascular effects including dyslipidemias and hypertension (Identifier no.: NCT01563770), and for angina and inflammation (Identifier no.: NCT02870764). Fufangdanshen tablets that contain salvianolic acid along with several other products such as ginsenosides are currently under clinical trial for vascular dementia (Identifier no.: NCT01761227). While these clinical applications for various, but primarily cardiovascular diseases, are important indicators for therapeutic application of salvianolic acid and plant containing such compounds, direct efficacy under dementia conditions are yet to be demonstrated. Unlike the Aβ drugs that failed under clinical trials, however, these compounds may have favourable outcome given their diverse mechanisms listed throughout this review.

## 7. Conclusions

Caffeic acid is a gift of nature that is commonly abundant in various food ingredients such as coffee, honey and fruits and vegetables. The dimerization of caffeic acid or conjugation with another phenolic acid, danshensu, through unique biosynthesis pathway in some plants such as sage have paved the way to identify potential therapeutic agents for dementia. Such compounds belonging to the salvianolic acid and RA derivatives have shown promising effect both in vitro and in vivo at doses that are considered very potent. Beyond the common antioxidant and anti-inflammatory mechanisms, specific effects on Aβ and tau protein pathologies coupled with potential recovery of neuronal loss from stem cells have been documented. All these pieces of evidence thus warrant further research, particularly clinical trials, that further validate their potential for treating dementia. The most significant effect that needs highlighting as a concluding remark is their potential in both AD and CVD cases of dementia.

## Figures and Tables

**Table 1:** In vitro neuroprotective effects of Salvianolic acids.

Compound	Model	Outcome	Reference
SA-B	LPS-stimulated primary microglial cells from mice	40 µM—Inhibit microglial activation; enhance neural precursor cell proliferation, differentiation, and survival; inhibit NF-κB activation along with ROS, NO, iNOS and cytokine (IL-1β, TNF-α and IL-6) production.	[29,30]
SA-A	SH-SY5Y cells treated with Aβ; Aβ aggregation assay	Cytoprotective; inhibit Aβ self-aggregation; disaggregates pre-formed fibrils; reduce metal-induced aggregation through chelating metal ions; reduce iROS.	[31]
SA-B	N2a-mouse and H4-human neuroglioma cell lines expressing SwedAPP cells	Decrease extracellular Aβ, soluble APPβ, and intracellular C-terminal fragment β from APP; no effect on α-secretase and γ-secretase activity and the levels of FL-APP; protein-docking model show interactions with the BACE1 catalytic centre.	[32]
SA-B	SH-SY5Y-APPsw cells	(25, 50, or 100 µM)—Reduce Aβ_1–40_ and Aβ_1–42_ level in culture media; decrease the protein expressions of BACE1 and sAPPβ; ADAM10 and sAPPα; inhibit GSK3β activity; attenuate oxidative stress (enhance SOD and GPx activities).	[33]
SA-B	Aβ_25–35_-treated PC12 cells	20 µM—Reverse the reduced expression level of BPRP; increase cell viability; reduce ROS and iCa^2+^.	[34]
SA-B	oxygen-glucose deprivation and reoxygenation (OGD/R) model in PC12 cells and primary cortical neurons	Ameliorate NeuN protein release; inhibit the TLR4/MyD88/TRAF6 signaling pathway; inhibit NF-κB transcriptional activity and pro-inflammatory cytokine (IL-1β, IL-6, and TNF-α).	[35]
SA-B	Primary culture of oligodendrocyte precursor cells from rats	20 μg/mL—promote differentiation.	[36]
SA-B	Aβ_25–35_-treated PC12 cells; enzyme assay	200 μg/mL—Revise cytotoxicity; Ca^2+^-intake and LDH release; inhibit AChE.	[37]
SA-B	Aβ_1–40_ fibril formation and destabilization; Aβ_1–40_-treated SH-SY5Y cells	Inhibit fibril aggregation (IC_50_: 1.54–5.37 µM); destabilize preformed Aβ fibril (IC_50_: 5.00–5.19 µM); inhibit cytotoxicity (above one μM).	[38]
SA-B	H_2_O_2_-treated PC12 cells	(0.1–10 µM)—Pre-treatment—Improve cell survival and activities of SOD, CAT and GPx; suppress MDA, LDH, iCa^2+^, caspase-3 activity and apoptosis.	[39]
Salvianolic acid of commercial source—undescribed	Primary astrocytes from rats—OGD-induced mitochondria damage	Cytoprotective and reverse ΔΨ(m) reduction	[40]
SA-B	Bone marrow derived neural stem cells	Induce BDNF production; protect cells from H_2_O_2_ toxicity; promote self-renewal and neuronal differentiation.	[41]
SA-B	Neural stem/progenitor cells	5–50 µM—Promote proliferation; up-regulate the expression of nestin; maintain self-renewal; effect mediated via PI3K/Akt pathway.	[42]
SA-B	OGD/RP-induced damage in primary rat cortical neurons	Enhance cell viability and the activities of Mn-SOD, CAT and GPx; elevate ΔΨ(m) (*p* < 0.01) and depress the release of cytochrome c; reverse neuronal morphological injury.	[43]
SA-B	NSCs from mice	20 and 40 µg/mL—Increase the number of NSCs and their derivative neurospheres; increase G2/S-phase cell population; promote neurite outgrowth, proliferation and differentiation of NSCs.	[44]
SMND-309 (see Figure 4)	Cultured rat cortical neuron under OGD	3–100 µM—Increase cell survival rate, mitochondrial antioxidant enzyme activities, respiratory enzymes activities, respiratory control ratio and ATP content; decrease mitochondrial MDA content, LDH release, iCa^2+^ level and caspase-3 activity.	[45]

Akt, protein kinase B; APP, Amyloid precursor protein; BACE1, beta-secretase 1; BDNF, brain-derived neurotrophic factor; BPRP, brain–pancreas relative protein; CAT, catalase; GFAP, Glial fibrillary acidic protein; GPx, glutathione peroxidase; Allograft inflammatory factor 1; iROS, intracellular reactive oxygen species; LDH, lactate dehydrogenase; ΔΨ(m) or mmp, mitochondrial membrane potential; MDA, malondialdehyde; MyD88, myeloid differentiation primary response 88; NF-κB, nuclear factor κB; NSCs, neural stem cells; OGD/RP, oxygen-glucose deprivation/reperfusion; PI3K, phosphatidylinositol-4,5-bisphosphate 3-kinase; sAPP, soluble APP; SA-A, salvianolic acid A; SA-B, salvianolic acid B; SOD, superoxide dismutase; TLR4, toll-like receptor 4; TRAF6, TNF receptor associated factor-6.

**Table 2:** In vivo neuroprotective effects of salvianolic acids.

Compound	Model	Outcome	Reference
SA-B	Ischemia/reperfusion injury model in mice—20, 40 or 60 mg/kg during reperfusion	Neuroprotective—decrease ROS level; suppress the expression of GFAP, Iba1, IL-1β, IL-6, TNF-α and cleaved-caspase 3; inhibit astrocytes and microglia overactivation.	[50]
Total salvianolic acid (commercial source)	APPswe/PS1dE mice model—30 and 60 mg/kg for 14 weeks	Improve learning and memory; decrease the LDL-C and cholesterol (higher dose) levels; decrease Aβ42 and Aβ40 levels in the hippocampus; increase glucose-6-phosphate, sucrose-6-phosphate, sorbitol, ascorbate (higher dose); reduce galactose and cholecalciferol in the hippocampus.	[51]
SA-A	Subarachnoid hemorrhage model in rats—10 or 50 mg/kg, i.p.	Reduce the elevated levels of ROS and MDA; increase GPx activity and GSH and BDNF in the cortex; decrease the release of inflammation cytokines (TNF-α, IL-1β, IL-6, and IL-8); reverse the decreased expression of Nrf2 and its downstream targets (HO-1 and NQO-1); No effect on phosphorylation of JNK but reversed the increased the phosphorylation of p38 MAPK and the decreased the phosphorylation of ERK.	[52]
SA-B	Vascular dementia model (permanent bilateral common carotid artery occlusion) in rats—20 mg/kg, p.o. for 6 weeks	Reverse the reduced hippocampal IGF-1 levels; increase phosphorylated-Akt level (Akt level not altered); inhibit apoptosis of hippocampal neurons in CA1 region.	[53]
Total Salvianolic acid	Angiogenesis and long-term neurological recovery after ischemic stroke—permanent distal middle cerebral artery occlusion—2 weeks treatment model.	Enhanced post-stroke angiogenesis, pericytes and astrocytic end feet covered ratio in the peri-infarct area; effects dependent on activation of JAK2/STAT3 signaling pathway.	[54]
SA-B	Chronic mild stress model in mice—20 mg/kg, i.p. for 3 weeks	Alter M1 microglial polarization toward M2 activation in the hippocampus and cortex; alleviate neuronal deficits in hippocampus; suppress pro-inflammatory markers (IL-1β, IFN-γ, IL-6 and iNOS,); reverse the decrease in IL-4 in both the hippocampus and the cortex; decrease the ratio of (IL-6^+^-Iba1^+^)/Iba1^+^ cells, and increased the ratio of (Arg-1^+^-Iba1^+^)/Iba1^+^ cells in hippocampus.	[29]
SA-B	Ischemia/reperfusion (I/R—transient middle cerebral artery occlusion) injury model in rats—3, 6 or 12 mg/kg, i.p.	Decrease I/R-induced neurological deficits, plasma-soluble P-selectin and soluble CD40 ligand, neuronal and DNA damage in the hippocampal CA1 region and neural cell loss in the ischemic core; inhibit mRNA and protein overexpression in the penumbra cortex, including ICAM-1, IL-1β, IL-6, IL-8, and MCP-1; reduce CD40 expression and NF-κB activation	[55]
Salvianolic Acids for Injections—crude mixture predominantly SA-B. (commercial source)	Ischemia/reperfusion or focal cerebral ischemia model—23 or 46 mg/kg, i.p. for 4 days—pretreatment	Decrease neuroinflammation and infarction volume; inhibit microglia activation along with TLR4/NF-κB-dependent release of cytokines (IL-1β and IL-6).	[56]
SA-A	Focal cerebral ischemia (transit middle cerebral artery occlusion mice) model in mice—1 or 5 mg/kg, i.p.	Ameliorate neuronal damage, neurological deficit and volume of infarction; inhibit eNOS uncoupling and calpain proteolytic activity; suppress peroxynitrite generation; increase AKT, FKHR and ERK phosphorylation.	[57]
SA-A	Blood-spinal cord barrier (BSCB) in spinal cord injury model in rats—2.5, 5 or 10 mg/kg, i.p.	Neuroprotective effect via the expression of microRNA-101 (miR-101) under hypoxia; increase Nrf2 and HO-1 expression; improve the recovery of neurological function.	[58]
SA-A	Ischemic brain injury model in mice—50 and 100 μg/kg, i.v.	Neuroprotective and preserves the BBB; reduce oxidative stress and apoptosis; promote endogenous neurogenesis; reverse the expression levels of DCX and Bcl-2; suppress NF-κB signaling and inflammation/nitrosative stress; promote neurogenesis-related protein expression by modulating GSK3β/Cdk5 activity; enhance the expression levels of β-catenin/DCX and Bcl-2 for neuroprotection.	[59]
Commercially available salvianolic acid—undescribed	Cerebral infarction of I/R (MCAO model)—10 mg/kg injection	Neuroprotection via antioxidant mechanism (increased SOD and suppressed MDA levels); upregulate mtCx43 through PI3K/AKT pathway.	[40]
SA-B	MCAO model	Prevent gross cerebral I/R injury.	[35]
SA-B	Rat model of contusion by heavy impact to induce spinal cord injury—20 mg/kg, i.p. for 8 weeks	Increase myelin sheath and the number of regenerating axons; restore neurological function; decrease caspase-3 expression in the spinal cord.	[36]
SA-B	MCAO model—25 mg/kg administrated twice	Cerebral-protective effect—reduce infarct volume, lower brain oedema; increase neurological scores; decrease TNF-α and IL-1β levels in brain tissue; upregulate the expression of SIRT1 and Bcl-2; downregulate the expression of Ac-FOXO1 and Bax; effects abolished by SIRT1 inhibitor (EX527).	[60]
SA-B	Mouse model of cerebral ischemia and reperfusion injury (bilateral carotid artery occlusion)—22.5 mg/kg	Decrease MDA content and NOS activity of the pallium; increase SOD activity and the total antioxidant capability of the pallium.	[61]
Total salvianolic acids (commercial source)	MCAO model in rats—1.67 mg/kg, i.p. administrated before reperfusion	Attenuate I/R-induced microcirculatory disturbance and neuron damage; activate AMPK, inhibit NADPH oxidase subunits membrane translocation, suppress Akt phosphorylation and PKC translocation.	[62]
SMND-309 (see Figure 4)	MCAO model in the rats	Decrease infract volume; improve neurological function and neuronal survival; promote angiogenesis by increasing the levels of erythropoietin (EPO), erythropoietin receptor (EPOR), phosphorylated JAK2 and STAT3, VEGF and VEGF receptor 2 (Flk-1) in the brain.	[63]
SA-A	Transgenic *Caenorhabditis elegans*	Inhibit Aβ-induced paralysis.	[31]
SA-B	Aβ_25–35_ injected intracerebroventricularly in mouse—10 mg/kg, p.o. for 7 days	Reverse memory impairment in the passive avoidance task; reduce microglia and astrocytes activation; reduce iNOS and COX-2 expression and TBRS level; restore ChAT and BDNF protein levels.	[64]
SA-B	Traumatic brain injury in mice in cortical impact model—25 mg/kg, i.v.	Reduce brain oedema, lesion volume and motor functional deficits; improve spatial learning and memory; inhibit the neutrophil infiltration and microglial activation; suppress the expression of pro-inflammatory cytokines (TNF-α and IL-1β) and enhance the expression of anti-inflammatory cytokines (IL-10 and TGF-β1) in brain tissues.	[65]
SA-B	Transient global ischemia in rats via irreversibly vertebral arteries occlusion—50 mg/kg, i.p. for 4 weeks	Protect learning and memory functions.	[42]
SA-B	Drug-induced amnesic models induced by scopolamine, diazepam, muscimol, or Aβ_25–35_—10 mg/kg, p.o.	Reverse cognitive impairments induced by scopolamine or Aβ_25–35_; Effect via the GABAergic neurotransmitter system.	[66]
SMND-309	MCAO model in rats—2.5, 5 or 10 mg/kg i.v. 3 and 12 h after occlusion	Decrease neurological deficit scores, reduce the number of dead hippocampal neuronal cells, mitochondria swelling and ROS production; mmp level and mitochondrial respiratory chain complex activities; at 25.0 mg/kg—neuroprotective effect still present 7 days after ischemia.	[67,68]
SA-B	Cerebral ischemia-reperfusion model in rats via carotid artery occlusion—10 mg/kg i.v.	Inhibit the decrease in SOD, GSH, and ATP levels and the increase in MDA and lactic acid levels.	[69]

AMPK, 5′ adenosine monophosphate-activated protein kinase; BBB, blood brain barrier; BSCB, blood-spinal cord barrier; ChAT, choline acetyltransferase, COX-2, cyclooxygenase 2; ERK, mitogen-activated protein kinases; FOXO1, Forkhead box protein O1 (FKHR) ; GFAP, glial fibrillary acidic protein; GSK, glycogen synthase kinase; HO-1, heme oxygenase-1; Iba1, ionized calcium-binding adapter molecule 1; IGF-1, insulin-like growth factor-1; iNOS, inducible nitric oxide synthase; Ionized calcium binding adaptor molecule 1; I/R, JAK2, Janus kinase 2, LDL, Low-density lipoprotein; MCAO, Middle cerebral artery occlusion; mtCx43, mitochondrial connexin 43; NADPH, Nicotinamide adenine dinucleotide phosphate (reduced form); Nrf2, erythroid 2-related factor 2; NOS, nitric oxide synthase; NQO-1, NAD(P)H dehydrogenase [quinone] 1, PKC, protein kinase C; SIRT1, sirtuin (silent mating type information regulation 2 homolog) 1; STAT3, Signal transducer and activator of transcription 3; TBRS, thiobarbituric acid reactive substances; TGF-β1, Transforming growth factor beta 1.
